# Current evidence on the management of re-recurrent rectal cancer: a systematic review

**DOI:** 10.3389/fonc.2026.1867185

**Published:** 2026-07-15

**Authors:** Georgios Giannos, Panagiotis Theodoropoulos, Maximos Frountzas, Sheng Qiu, Maria Katsigeorgis, Sophia Y. Chen, Shahnawaz Rasheed, Paris Tekkis, Bashar Safar, Christos Kontovounisios

**Affiliations:** 1 2nd Surgical Department, Hygeia Hospital, Athens, Greece; 2 Department of Colorectal Surgery, Royal Marsden Hospital, London, United Kingdom; 3 Division of Colon and Rectal Surgery, Department of Surgery, NYU Grossman School of Medicine, NYU Langone Health, New York, NY, United States; 4 Department of Surgery and Cancer, Imperial College, London, United Kingdom; 5 Department of Colorectal Surgery, Chelsea and Westminster Hospital, London, United Kingdom

**Keywords:** plastic-reconstruction, re-chemotherapy, re-irradiation, re-recurrent pelvic cancer, re-recurrent rectal cancer, re-resection, second recurrence, survival

## Abstract

**Background:**

Re-recurrent rectal cancer (RRRC) represents a highly complex disease following curative-intent treatment of recurrent rectal cancer (RRC). While management principles for primary and locally recurrent rectal cancer (RRC) have been defined by expert collaborations, no specific guidelines currently outline the perioperative management of RRRC. This systematic review aimed to appraise the reported perioperative strategies and oncological outcomes of patients undergoing curative-intent treatment for RRRC.

**Methods:**

Eligibility criteria, Studies reporting perioperative management and outcomes of adult patients undergoing curative-intent treatment for RRRC were included. Non-English articles, letters, abstracts, and studies lacking surgical or oncological data were excluded. Information sources, The review was conducted according to PRISMA guidelines and registered in PROSPERO (CRD420251244390). MEDLINE (PubMed), Cochrane Library, Web of Science, and Scopus were searched for relevant articles. Risk of bias, Methodological quality was assessed using the Newcastle–Ottawa Scale for cohort studies. Synthesis of results, Given heterogeneity in treatment strategies and outcome reporting, results were synthesized narratively.

**Results:**

Included studies, Three retrospective cohort studies comprising 169 patients treated with curative intent surgery for RRRC were included. Synthesis of results, Neoadjuvant therapy was administered in 20–92% of included patients, depending on the previous cumulative radiation dose. Pelvic exenteration was frequently required, with total exenteration performed in 6–20% and sacrectomy in up to 15% of cases; reconstructive procedures were reported in less than 16%. IORT was used in 44–77% of patients in centers where it was available. R0 resection rates ranged from 33% to 62%, with oncological outcomes directly associated with margin status.

**Discussion:**

Limitations of evidence, Evidence was limited to retrospective observational studies with small sample sizes, heterogeneous management, and varied institutional resources, precluding meta-analysis. Interpretation, Curative-intent surgery for RRRC is feasible in highly selected patients, with R0 being the principal prognostic determinant of oncological outcome. However, significant variability in perioperative pathways, margin definition, MRI-based classification, and reconstructive strategies underlines the necessity for the development of standardized, consensus-based recommendations to optimize multidisciplinary treatment.

**Systematic Review Registration:**

https://www.crd.york.ac.uk/PROSPERO/view/CRD420251244390, identifier CRD420251244390.

## 1 Introduction

Colorectal cancer is the third most commonly diagnosed malignancy and the second cause of cancer-related mortality worldwide (1). Despite advances in multimodal therapy, approximately 10% of patients treated for rectal cancer will develop local recurrence after curative-intent treatment (2). Up to 60% of patients with recurrent rectal cancer may suffer from subsequent pelvic recurrence within the 5-year postoperative period (3).

Re-recurrent rectal cancer (RRRC) constitutes a complex disease requiring specialized expertise and advanced experience in redo pelvic surgery, re-irradiation strategies, and reconstruction techniques. While the management of primary rectal cancer or local recurrent rectal cancer has been addressed by consensus groups, including beyond-TME (bTME) collaboration, no specific guidelines outline the perioperative management of RRRC (4).

Given the limited evidence and absence of structured recommendations, a comprehensive analysis of current literature is warranted. The main purpose of the present review was to systematically appraise the reported perioperative management of curative-intent RRRC, including anatomical tumor characteristics, neoadjuvant and re-irradiation strategies, extent of pelvic exenteration and reconstructive strategies, resection margins, and associated outcomes. These endpoints may indicate areas where further standardization and consensus-based recommendations are needed, offering insights for future treatment strategies.

## 2 Materials and methods

This systematic review was designed and conducted according to the Preferred Reporting Items for Systematic Reviews and Meta-analyses (PRISMA) guidelines (5). The protocol of the current review was registered in the PROSPERO (International Prospective Register of Systematic Reviews) database (CRD420251244390).

### 2.1 Search strategy and data handling

Two independent researchers (G.G., M.F.) identified eligible studies by screening MEDLINE (PubMed), Cochrane Library, Web of Science and Scopus databases. The final search was performed on 10 December 2025. The full strategy, including the list of search terms, Boolean operators, and database-specific search syntax, is provided in **Supplementary Table 1**.

Extracted data were tabulated using a pre-established data extraction form developed in Microsoft Excel. Two researchers (G.G., M.F.) independently screened titles and abstracts to identify potential eligible studies. The full texts of selected articles were further assessed independently by the same reviewers for fulfillment of predefined inclusion criteria. Discrepancies in the literature search process and data extraction were resolved by a third reviewer (C.K.). A further review of the reference lists was conducted to ensure that all eligible studies were included.

Records for which the full text could not be retrieved were excluded and reported in the PRISMA flow diagram.

No contact with corresponding authors was made for additional information; therefore, the analysis was limited to data reported in the original publications.

### 2.2 Inclusion and exclusion criteria

Recurrent rectal cancer (RRC) was defined according to the Beyond TME consensus as recurrence, progression, or development of new sites of rectal tumor within the pelvis after previous resectional surgery for rectal cancer. Re-recurrent rectal cancer (RRRC) was defined as a second pelvic recurrence occurring after previous treatment of RRC, consistent with the definitions reported in the included studies. We included studies that reported data on the management of adult patients with a second recurrence of rectal cancer. Eligible study types included randomized controlled trials, cohort studies, systematic reviews, case series, and case reports.

Abstracts, book chapters, editorials, letters, and studies published in non-English language were excluded, which may represent a potential source of language bias. Studies that did not provide information regarding the surgical and oncological management either pre- or post-operatively were not included. As imaging and histopathological evaluation are vital for the treatment strategy, studies lacking these data were also excluded.

### 2.3 Endpoints

Primary endpoints were data related to perioperative management of curative-intent RRRC: i) demographics, ii) period between treatment of RRC and RRRC appearance, iii) anatomical distribution of the tumor, iv) presence of metastatic disease, v) type of neoadjuvant and adjuvant therapies, vi) the frequency and type of pelvic exenteration, vii) performance of bone excision and plastic reconstruction, viii) administration of intraoperative radiation therapy (IORT) or hyperthermic intraperitoneal chemotherapy (HIPEC), ix) resection margin status, and x) survival outcomes (overall survival [OS] and progression-free survival [PFS]).

Secondary endpoints included relevant data associated with the perioperative treatment of RRC in the same patients who subsequently developed RRRC within the included cohorts. These data were extracted and presented separately from the primary RRRC outcomes to provide information regarding disease progression from RRC to RRRC.

### 2.4 Risk of bias assessment

The quality of included studies was assessed independently by two reviewers (G.G., M.F.) using the Newcastle–Ottawa Scale (NOS) for cohort studies (6). Any discrepancies were resolved by consensus with a third reviewer (C.K.). The NOS is a comprehensive tool used to appraise the risk of bias based on the following domains i) representativeness of the exposed cohort, ii) selection of the non-exposed cohort, iii) ascertainment of exposure, iv) demonstration that the outcome of interest was not present at the start of the study, v) comparability of cohorts on the basis of design or analysis, vi) assessment of the outcome, vii) whether follow-up was long enough for outcomes to occur, and viii) adequacy of follow-up of cohorts. All domains can be scored with one star, except the comparability section, which can be evaluated with a maximum of two stars. Each question was answered numerically ‘‘0’’ or ‘‘1’’, indicating whether the criterion was satisfied. Study scores of 7–9 indicate low risk of bias, 4–6 moderate, and 0–3 high risk of bias. Study scores of 7–9 were considered low risk of bias, scores of 4–6 indicated moderate risk, and scores of 0–3 were suggestive of high risk of bias.

### 2.5 Reporting bias assessment

Reporting bias assessment was initially planned to be performed. However, due to the lack of pre-published study protocols, the limited number of included studies, and substantially heterogeneity, no application of formal tool analysis was feasible.

### 2.6 Certainty of evidence

Assessment of the certainty of evidence using GRADE system was not able to be performed because of the limited number of studies and substantial heterogeneity (7). Hence, the certainty of evidence was evaluated qualitatively according to study design, risk of bias, consistency and precision of the reported outcomes.

### 2.7 Data synthesis

Given the heterogeneity of treatment strategies and reported outcomes, quantitative synthesis and meta-analysis could not be performed. Results were synthesized narratively and reported as presented in the original studies. In particular, data were descriptively presented and structured by outcome domain to allow a systematic appraisal of the findings.

## 3 Results

### 3.1 Study selection

**Figure 1:**
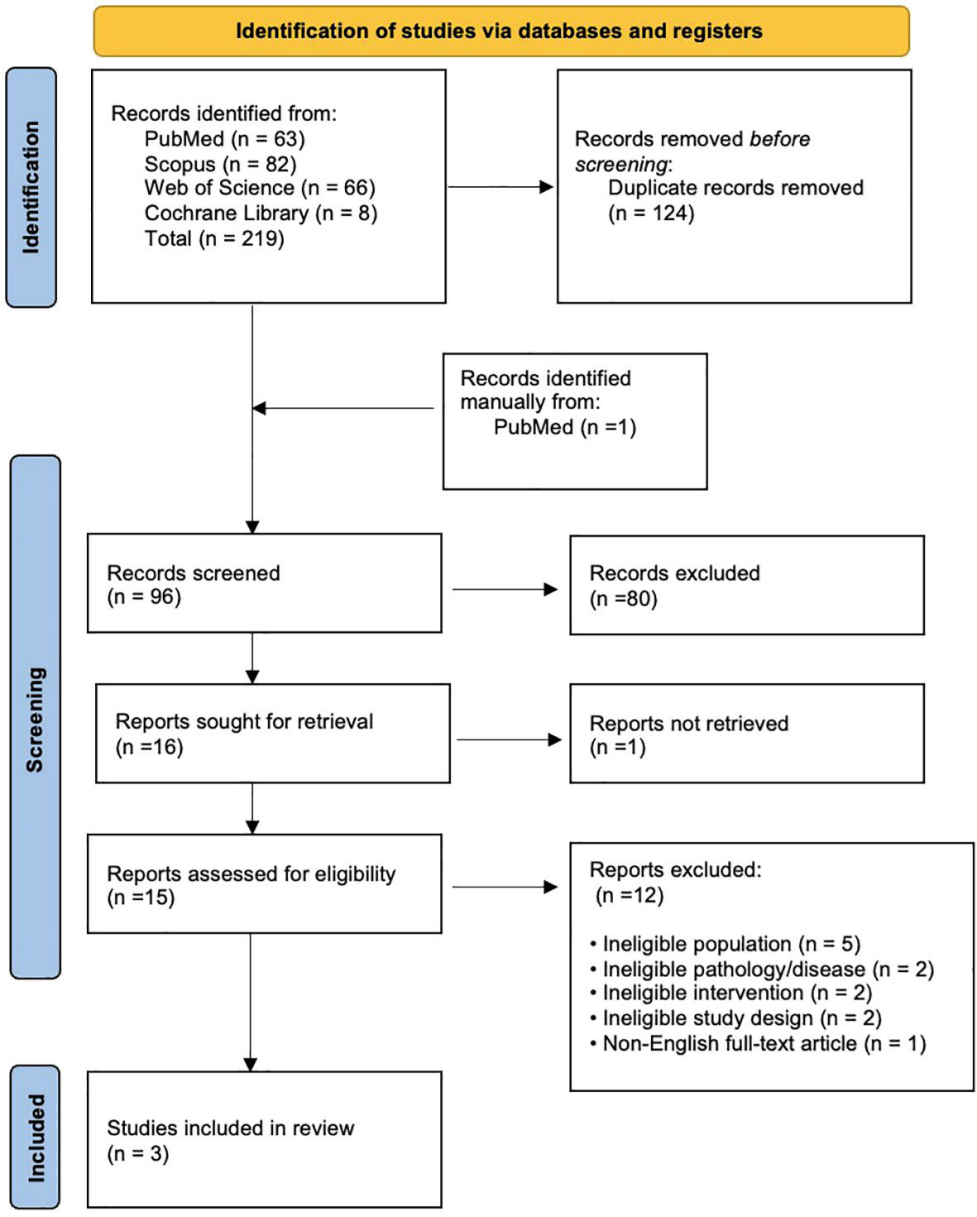
PRISMA flow diagram illustrating the study selection process for inclusion in the systematic review.

The initial literature search yielded 219 publications. Following a manual exclusion of duplicate records and the manual addition of a relevant study from PubMed (8), 96 records remained for further screening. Title/Abstract screening resulted in the exclusion of 80 clearly irrelevant studies. Of the remaining 16 records, one study was excluded because its full text was not available (9). Following full text investigation of the remaining 15 publications, 3 studies met the eligibility criteria for inclusion in this systematic review (3, 10, 11). **Figure 1** summarizes the study selection flow diagram.

### 3.2 Study characteristics

We identified a total of 169 cases of RRRC treated with curative-intent. All three eligible studies were retrospective cohort studies conducted in three different countries. Harji et al. in the United Kingdom collected data on 30 patients who underwent surgical management for second recurrence of rectal cancer between 2001 and 2010 at their institution (10). Colibaseanu et al. in the United States included 47 patients who had reoperative surgery in their department following failure of treatment for RRC between 1997 and 2007 (11). Recently, Creemers et al. published the outcomes of 277 patients with RRRC at two tertiary referral centers in the Netherlands between 1994 and 2022; of these 277 cases, only 92 patients were treated with curative-intent and were therefore included in the present review (3).

All relevant data are presented in detail in **Tables 1** and **2**, describing the curative-intent management of RRRC and RRC, respectively.

**Table 1:** Characteristics, management, and outcomes of curative-intent cohorts with re-recurrent rectal cancer.

Study	Country	Study design	Sample size (M/F)	Median age	Median time between RRC and RRRC (months)	Compartment	Multifocal RRRC	Distant metastasis	Neoadjuvant treatment	Pelvic exenteration	Bone resection	Reconstruction	IORT	HIPEC	R-status	Adjuvant treatment	Overall survival
Harji et al., 2013 (10)	UK	Retro-spective	30 (21/9)	60 (46-70)	13	Anterior: 3 (10%) Central: 5 (17%) Posterior: 3 (10%) Side wall: 19 (63%)	NR	None	CT: 4 (13%) RT: 1 (3%) CRT: 1 (3%) None: 24 (80%)	Total: 5 (17%) ‡Anterior/ Posterior: Reported/ Not specified	†Sacrum: Reported/Not specified	NR	None	NR	R0: 10 (33%) R1: 15 (50%) R2: 5 (17%)	CT: 7 (23%) None: 23 (77%)	1-year: 77% 3-year: 27%
Colibaseanu et al., 2013 (11)	USA	Retro-spective	47 (31/16)	57 (30-84)	29	NR	NR	NR	RT: 33 (70%)	Total: 3 (6%) Anterior: 5 (11%) Posterior: 13 (28%)	Sacrum: 7 (15%) Coccyx: 1 (2%)	Rectus myocutaneous: 7 (54%) Gracilis: 2 (15%) Pedicled omental flaps: 4 (31%)	36 (77%)	None	R0: 28 (60%) R1: 15 (32%) R2: 4 (9%)	NR	2-year: 83% 5-year: 33%
*Creemers et al., 2025 (3)	Nether-lands	Retro-spective	92 (47/45)	64 (SD 10)	14	Anterior: 18 (20%) Central: 20 (22%) Posterior: 24 (26%) Side wall: 30 (33%)	Yes: 27 (29%) No: 54 (59%) Missing: 11 (12%)	Yes: 6 (7%) No: 84 (91%) Missing: 2 (2%)	Induction CT: 43 (47%) CRT: 49 (53%) Induction CT + CRT: 7 (8%) None: 7 (8%)	Total: 16 (20%) Posterior: 4 (5%)	NR	NR	36 (44%)	4 (4%)	R0: 50 (62%) R1: 27 (33%) R2: none mis-sing: 4 (5%)	NR	3-year: 48%

* Of 92 patients initially considered for curative-intent treatment, 85 received neoadjuvant treatment and 81 underwent surgical operation.

‡ Anterior/Posterior exenteration was reported as part of overlapping procedures using “±” notation, without quantitively reporting the exact frequency.

† Sacrectomy or bony pelvic resection was reported as part of overlapping procedures using “±” notation, without quantitively reporting the exact frequency.

RRRC, Re-Recurrent Rectal Cancer; RRC, Recurrent Rectal Cancer; IORT, Intraoperative Radiation Therapy; HIPEC, Hyperthermic Intraperitoneal Chemotherapy; NR, Not Reported; CT, Chemotherapy; CRT, Chemoradiotherapy; RT, Radiotherapy; SD, Standard Deviation.

**Table 2:** Characteristics, management, and outcomes of curative-intent cohorts with recurrent rectal cancer.

Study	Country	Study design	Sample size (M/F)	Median time between primary surgery and RRC (months)	Compartment	Multifocal RRC	Metastasis	Neoadjuvant treatment	Pelvic exenteration	Bone resection	Reconstruction	IOPR	HIPEC	R-status	Adjuvant treatment
Harji et al., 2013 (10)	UK	Retro-spective	30 (21/9)	30	Central: 13 (43%) Sacral: 5 (17%) Side wall: 4 (13%) Central + Side wall: 6 (20%) *Composite: 2 (7%)	NR	Yes: 3 (10%) No: 0 (0%)	RT: 1 (3%) CRT: 12 (40%) None: 17 (57%)	Total: 2 (7%) Anterior: 3 (10%) Posterior: NR	†Sacrum: Reported/Not specified	NR	None	NR	R0: 15 (50%) R1: 13 (43%) R2: 2 (7%)	CT: 4 (13%) RT: 3 (10%) Brachy-therapy: 1 (3%) None: 22 (73%)
Colibaseanu et al., 2013 (11)	USA	Retro-spective	47 (31/16)	NR	NR	NR	NR	NR	NR	Sacrum: 3 (6%) Coccyx: 2 (4%)	NR	26 (55%)	None	R0: 42 (89%) R1: 5 (11%) R2: 0 (0%)	NR
‡Creemers et al., 2025 (3)	Nether-lands	Retro-spective	92 (47/45)	34	Anterior: 12 (13%) Central: 45 (49%) Posterior: 16 (17%) Side wall: 21 (19%)	13 (14%)	Yes: 15 (17%) No: 72 (83%)	induction CT: 15 (16%) CRT: 60 (65%) induction CT + CRT: 57 (62%)	Total: 1 (1%) §Posterior: Reported/Not specified	§Sacrum: Reported/Not specified	NR	44 (40%)	4 (4%)	R0: 45 (62%) R1: 36 (33%) missing: 11 (5%)	NR

*According to the Leeds classification, composite recurrences involve combined sacral and pelvic side-wall structures.

† Sacrectomy or bony pelvic resection was reported as part of overlapping procedures using “±” notation, without quantitively reporting the exact frequency.

‡ Of 92 patients initially considered for curative-intent treatment, 75 received neoadjuvant treatment.

§Posterior exenteration was grouped together with abdominosacral resection, without reporting the exact number of cases.

RRC, Recurrent Rectal Cancer; NR, Not Reported; IORT, Intraoperative Radiation Therapy; HIPEC, Hyperthermic Intraperitoneal Chemotherapy; CT, Chemotherapy; CRT, Chemoradiotherapy; RT, Radiotherapy.

### 3.3 Management of RRRC (second recurrence)

#### 3.3.1 Preoperative assessment

All RRRC cases were discussed within their institutional multidisciplinary team (MDT) meetings, formulating individualized treatment plans. Each center followed its own protocol as for the preoperative management. All institutions used diagnostic modalities such as serum Carcinoembryonic Antigen (CEA) levels, Computed Tomography (CT) of the thorax, abdomen and pelvis, pelvic magnetic resonance imaging (MRI) for assessment of locoregional disease, and positron emission tomography-CT (PET-CT) for detection of possible distant metastatic disease. Although histological confirmation was a part of the preoperative work-up, it was not always feasible or necessary in the included cohorts. One study reported that biopsy was obtained in 27 of 30 cases (90%) before surgery, while in another cohort, CT-guided biopsy was routinely performed preoperatively, with only 1 (2%) patient proceeding to surgery without histopathological confirmation due to technical reasons. In those circumstances, the diagnosis was based on CEA levels and imaging studies (10, 11). In the third study, treatment decisions were guided by the combined assessment of imaging and tumor marker CEA levels and/or biopsy, although the portion of patients who underwent tissue sampling was not specified (3).

#### 3.3.2 Eligibility criteria for curative-intent surgery

The main criteria for considering a patient candidate for curative intent surgery were the anticipated ability to achieve clear margins (R0), performance status, and the absence of unresectable metastatic disease. In one study, high sacral involvement (S1-S2), extensive pelvic sidewall involvement, multifocal disease, encasement of major vessels (aorta and iliac vessels), and protrusion to sciatic notch were defined as absolute contraindications (10). In contrast, another study appraised involvement of the sheath of major vessels, sacral (S1-S2) or lumbar invasion, and unilateral pelvic sidewall disease requiring lower extremity amputation as relative contraindications (11). In the third study, the authors stated more generally that advanced unresectable disease and poor performance status were indications for palliative treatment (3). Resectable metastatic disease was considered a relative contraindication across all studies.

#### 3.3.3 Demographics and timing of re-recurrence

The gender distribution and median age of patients with RRRC were broadly similar across the included studies. In the smallest cohort, 70% of patients were male, with a median age of 60 years (range 46 -70) (10). Another study had 66% of patients who were male with a median age of 57 years (range 30 - 84) (11). The largest study reported 51% male patients with a median age of 64 years (SD 10) (3).

Regarding the interval between treatment of RRC and diagnosis of RRRC, the median time varied among the three studies and were reported as 13, 29, and 14 months, respectively.

#### 3.3.4 Affected compartments, multifocality and metastatic disease

In the study with the largest cohort, RRRC most frequently developed in the pelvic sidewall (33%), followed by the posterior (26%), central (22%), and anterior (20%) compartments. Multifocal disease was detected in 29% of cases, with missing data in 12%. Distant metastatic disease was identified in 7% of patients, with 2% missing data (3).

In the smallest cohort, the most common site of recurrence was the pelvic sidewall (63%), whereas central, anterior and posterior compartments were affected in 5%, 3%, 3% of cases, respectively. None of the patients in this cohort had distant metastatic disease at the time of the second recurrence. Although multifocal disease was considered a contraindication, the authors did not report whether any patient with resectable disease had multiple foci (10).

Data on compartment distribution and the presence of multifocal or distant metastatic disease were not reported in the third study (11).

#### 3.3.5 Neoadjuvant treatment

Each cohort study followed their own institutional protocol, resulting in heterogeneity in therapeutic selection. In the largest cohort, 85 (92%) patients received neoadjuvant treatment based on previous oncological treatment. Neoadjuvant chemoradiotherapy (CRT) was administered to 49 (53%) patients, of whom 19 (40%), 1 (2%) and 25 (51%) received doses of 50-50.4 Gy, 5x5 Gy and 30–30 Gy, respectively. Four of 49 patients (8%) were treated with alternative regimens. Induction chemotherapy (CT) was provided to 43 (47%) cases, while 7 (8%) received both induction CT and subsequent CRT (3).

The institutional protocol of the second study recommended the addition of external radiotherapy (20–30 Gy) preoperatively in cases where the cumulative radiation dose for treatment of the primary tumor or first recurrence was less than 64 Gy. Accordingly, 33 patients (70%) received RT (11).

In the third study, the majority of patients (80%) underwent surgery directly without preoperative treatment (10).

CT, RT and CRT were administered to 4 (13%), 1 (3%) and 1 (3%) case, respectively.

#### 3.3.6 Extent of surgical resection and plastic reconstructive management

Pelvic exenteration is one of the most common procedures for achieving radical margins for curative-intent RRRC, often requiring bone resection or plastic surgery reconstruction.

In the largest study, 81 of 92 patients (88%) were deemed eligible for curative-intent resection after completing neoadjuvant treatment and imaging reassessment. 16 (20%) patients underwent total exenteration, while posterior exenteration was performed in 4 (5%) cases. However, no data regarding bone resection and reconstructive strategies were reported (3).

Another study mentioned that total, anterior, and posterior exenteration were carried out in 3 (6%), 5 (11%), and 13 (28%) patients, respectively. Sacral resection was required in 7 (15%) cases, and coccyx was removed in 1 (2%) patient. Soft tissue reconstruction was performed in 13 (16%) cases, most commonly using rectus abdominis myocutaneous (RAM) flap in 7 of 13 cases (54%), followed by omental flap (31%) and gracilis muscle flap (15%) (11).

The third study noted that total exenteration was performed in 5 (17%) cases; the exact numbers of anterior or posterior exenteration or whether sacral excision was performed were not specified. No data were reported on plastic surgery reconstruction (10).

#### 3.3.7 Intraoperative radiation therapy and hyperthermic intraperitoneal chemotherapy

In one study, IORT was not available at the center, and the use of HIPEC was not reported (10).

According to the institutional protocol of the second study, IORT was delivered in situations where margins were less than 5 mm, frozen-section margins were positive, or negative margins were difficult to achieve. A total of 36 of 47 patients (77%) received IORT, of whom 20 had R0 resections and 15 had R1 resections. None of the patients in this cohort underwent HIPEC (11).

In the largest cohort, IORT administered to 36 (44%) patients, including 11 had R1 margin status. In addition, 4 (4%) cases received HIPEC during cytoreductive surgery (3).

#### 3.3.8 Resection margin status

**Figure 2:**
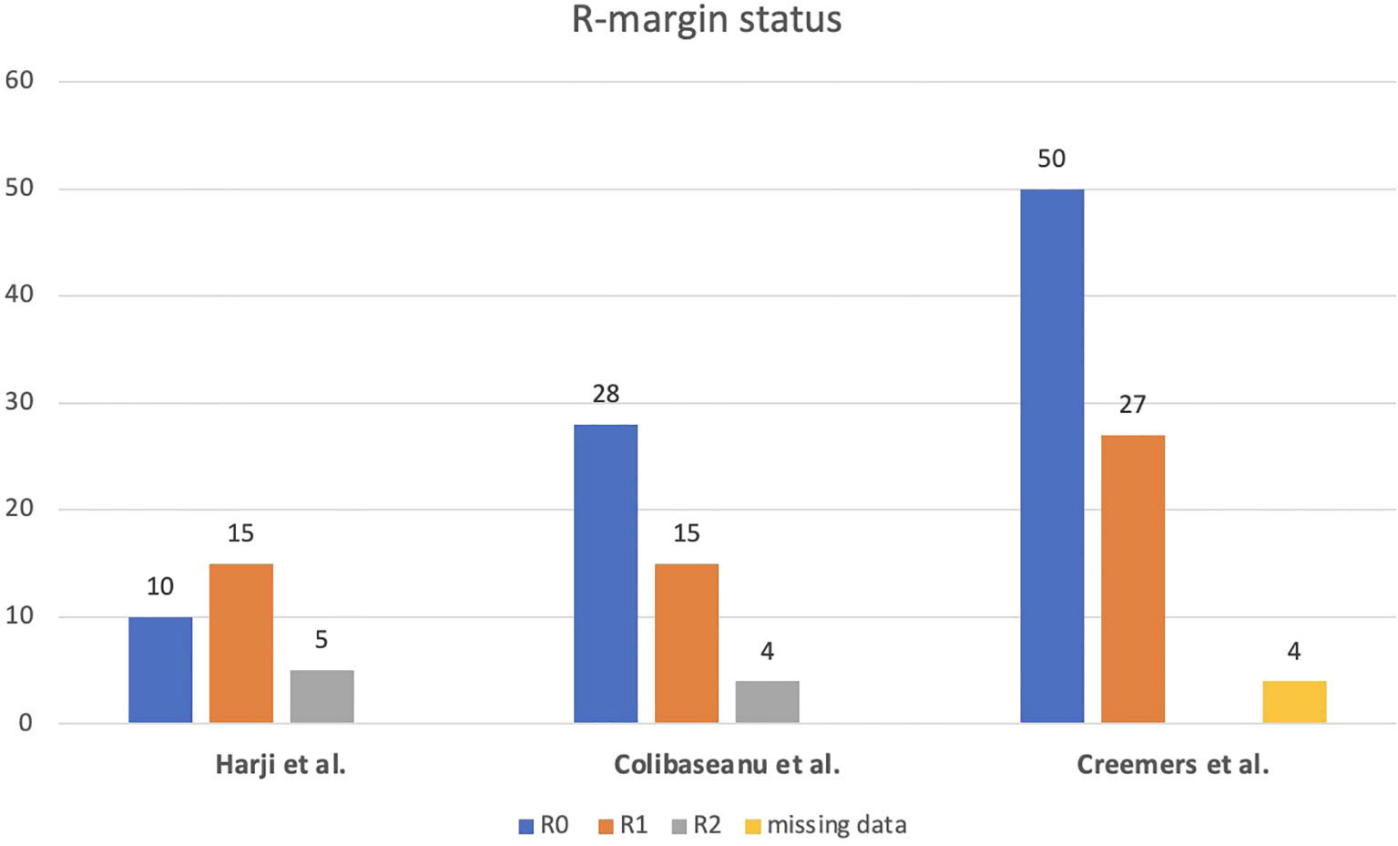
Resection margin status following curative-intent surgery for RRRC.

Resection margin status following curative-intent surgery for RRRC varied across cohorts, as demonstrated in **Figure 2**.

In the smallest study, R0 resection was achieved in 10 (33%) patients, while 15 (50%) and 5 (17%) had R1 and R2 resections, respectively (10). Histopathology reports from the second study revealed R0 margins in 28 (60%) cases, R1 in 15 (32%) and R2 in 4 (9%) patients (11).

In the largest cohort, R0 was obtained in 50 (62%), whereas R1 was reported in 27 (33%). No biopsy was indicative of R2 resection, while data on margin status were missing in 4 (9%) patients (3).

#### 3.3.9 Adjuvant treatment

Postoperative treatment strategies were reported in only one of the included studies. In which 7 of 30 patients (23%) received adjuvant chemotherapy. However, no specific regimen was further detailed (10).

#### 3.3.10 Oncological outcomes

**Figure 3:**
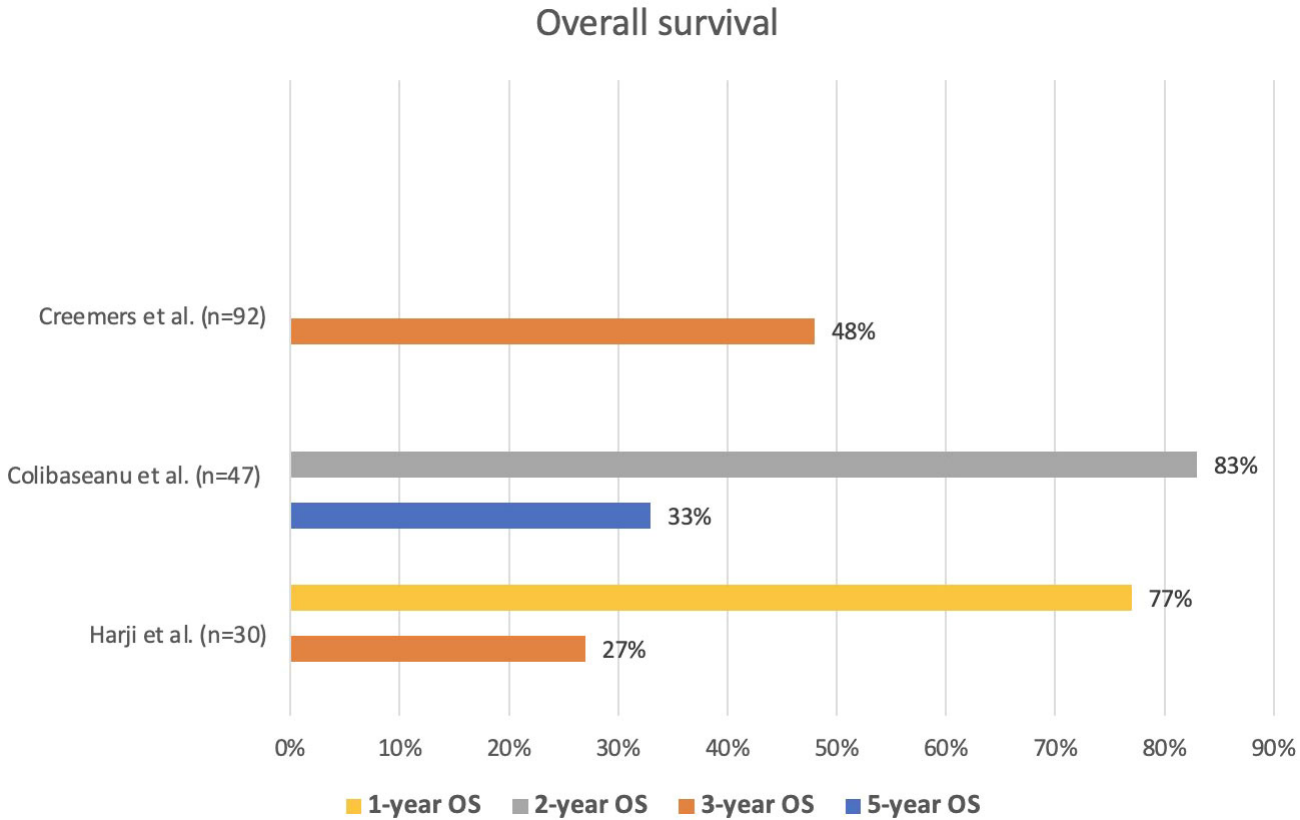
Overall survival following curative-intent surgery for RRRC.

Overall survival for patients undergoing curative-intent surgery for RRRC were reported at different time intervals as illustrated in **Figure 3**.

In the smallest cohort, OS at 1 year was 77% and subsequently declined to 27% at 3 years. Median survival following R0 resection was 32 months, compared with 19 months after R1 resection and 7 months after R2 resection (p=0.05). Median disease-free survival (DFS) was 14 months in patients who achieved R0 resection, 9 months after R1 resection, and 5 months after R2 resection (p = 0.196) (10).

The second study reported OS rates of 83% and 33% at 2 and 5 years, respectively. R0 resection was associated with 37% 5-year OS, R1 resection with 42%, and R2 resection with less than 2-year survival (p = 0.002). Median time to a third recurrence was 42 months following R0 resection, 19 months after R1 resection, and 11 months after R2 resection (p<0.001) (11).

In the largest study, 3-year OS was 48%. Median progression-free survival (PFS) was 19 months in patients with R0 margins and 9 months after R1 resection. Median local re-re-recurrence-free survival was 15 months following R0 resection and 7 months after R1 resection (3).

### 3.4 Management of RRC (first recurrence)

#### 3.4.1 Demographics and timing of recurrence

The cohorts treated for RRRC represent the same patients previously managed for RRC. Therefore, the number of patients and gender distribution are identical.

The median interval between the surgery for the primary tumor and the first recurrence was 30 months in the smallest study and 34 months in the largest cohort (3, 10). In the third study, the time from the initial operation to tumor relapse was not reported (11).

#### 3.4.2 Affected compartments, multifocality and metastatic disease

In the smallest study, recurrence was most frequently detected in central compartment (43%), followed by a combined central – sidewall pattern of invasion (20%), sacral (17%), and pelvic sidewall (13%) compartments. According to Leeds classification (12), 7% of patients presented with composite (sacral – sidewall) recurrence. At the time of diagnosis, 3 (10%) patients were found to have concurrent resectable metastatic disease. The presence of multifocal disease was not reported (10).

In the largest study, involved compartments were central (49%), pelvic sidewall (19%), posterior (17%), and anterior (13%). Distant metastases were reported in 15 (17%) patients, while multifocal disease was identified in 13 (14%) cases (3).

No data relevant to anatomical distribution or extent of RRC were provided in the third study (11).

#### 3.4.3 Neoadjuvant treatment

In the largest study, 75 of 92 patients (82%) received neoadjuvant treatment. Of these, 15 (16%) were treated with induction CT, 60 (65%) underwent CRT, and 57 (62%) received induction CT followed by CRT (3).

In the smallest cohort, RT and CRT were administered in 1 (3%) and 12 patients (40%), respectively (10).

Data regarding neoadjuvant treatment were unavailable in the remaining study (11).

#### 3.4.4 Extent of surgical resection and plastic reconstructive management

In the largest study, total exenteration was performed in 1 (1%) patient, whereas posterior exenteration or abdominosacral was carried out in 6 (7%), without specifying the exact number of each procedure (3).

For the second study, sacral excision was performed in 3 (6%) of cases and coccygectomy in 2 (4%) of patients. However, no additional data regarding pelvic exenteration were provided (11).

In the smallest cohort, total and anterior exenteration were performed in 2 (7%) and 3 (10%) patients, respectively, whereas sacrectomy was reported as part of the procedures without recording the exact number (10).

Reconstruction management was not addressed in any of the included studies.

#### 3.4.5 Intraoperative radiation therapy and hyperthermic intraperitoneal chemotherapy

IORT was available in two centers and was administered to 44 (40%) and 26 (55%) patients in the respective cohorts. In the same studies, HIPEC was delivered to 4 patients (4%) in one study and to none in the other (3, 11).

In the smallest study, IORT was not available and no data regarding HIPEC treatment were documented (10).

#### 3.4.6 Resection margin status

The largest cohort reported R0 resection in 45 (62%) patients and R1 in 36 (33%) patients, with missing data in 11 (5%) of the included population (3).

In the smallest study, R0 margins were achieved in 15 (50%) patients, while 13 (43%) and 2 (7%) had R1 and R2 resections, respectively (10).

In the remaining study, a higher rate of complete resection was observed, with R0 margins obtained in 42 (89%) cases. R1 resection was identified in 5 (11%) patients, and no R2 resections were detected (11).

#### 3.4.7 Adjuvant treatment

Postoperative management was reported in only one study. CT was provided to 4 (13%) patients, while RT was administered to 3 (10%) cases and brachytherapy to 1 (3%) patient. (3%) (10).

### 3.5 Risk of bias

The summary of the risk of bias is illustrated in **Table 3**. The overall bias was moderate (6/9) for two of the included studies due to the absence of comparator groups and multivariable adjustment. The largest study was deemed to have a low risk of bias, fulfilling most criteria. No study was judged to have a high risk of bias.

**Table 3:** Quality assessment template for cohort study based on Newcastle-Ottawa-Scale (NOS).

Study number	Author	Year	Selection bias assessment (maximum 4 stars)				Comparability (maximum 2 stars)	Outcome (maximum 3 stars)			Total score
			Representativeness of the exposed cohort	Selection of the non-exposed cohort	Ascertainment of exposure	Demonstration that outcome of interest was not present at start of study	Comparability of cohorts on the basis of the design or analysis	Assessment of the outcome	Was follow-up long enough for outcomes to occur	Adequacy of follow up of cohorts	
			Score	Score	Score	Score	Score	Score	Score	Score	
1	Harji et al. (10)	2013	1	0	1	1	0	1	1	1	**6**
2	Colibaseanu et al. (11)	2013	1	0	1	1	0	1	1	1	**6**
3	Creemers et al. (3)	2025	1	1	1	1	1	1	1	1	**8**

Risk of bias was assessed using the Newcastle–Ottawa Scale (NOS) for cohort studies, which includes three domains: selection (maximum 4 points), comparability (maximum 2 points), and outcome (maximum 3 points), with a total score ranging from 0 to 9. Higher scores indicate lower risk of bias. In each item, a score of “1” indicates that the criterion was satisfied, while “0” indicates that the criterion was not satisfied. Bold values represent the total NOS score.

### 3.6 Reporting bias

Assessment of the risk of reporting bias was not feasible to be performed due to the limited number of included studies. The absence of pre-published protocol and heterogeneity in outcome reporting precluded further evaluation of potential selective outcome reporting or endpoint discrepancies.

### 3.7 Certainty of evidence

The certainty of evidence was not systematically appraised using the GRADE framework because of the small number of included studies (7). However, overall confidence in the evidence was assessed due to the retrospective observational design, heterogeneity in patient selection, perioperative treatment strategies and outcome reporting across the included cohorts.

## 4 Discussion

This is the first systematic review appraising both the overall management of RRRC preoperatively, intraoperatively and postoperatively, and the impact of the respective treatment strategy or procedure. Given the absence of management guidelines for patients with RRRC, we decided to compare the reported therapeutic approach for RRRC patients of the included institutions with the beyond TME consensus statement for the patients with locally advanced rectal cancer or RRC (4).

### 4.1 Diagnosis & preoperative imaging

Tissue biopsy is the gold standard for the diagnosis of RRC or locally advanced rectal cancer. Patients with recurrent tumors unamenable to biopsy are recommended to undergo curative-intent surgery only if disease progression is confirmed by serial imaging (MRI pelvis, PET-CT) in combination with elevated CEA levels. The majority of RRRC were diagnosed with biopsy obtained either endoscopically or under CT guidance. In cases where histopathological confirmation was not feasible, the decision to proceed with surgery was made based on imaging findings and tumor marker CEA levels.

MDT meetings are the cornerstone of appropriate oncological management, assessing pelvic MRI, CT thorax-abdomen-pelvis, PET-CT, and CEA levels (4).

All included institutions were aligned with the aforementioned diagnostic workup (3, 10, 11).

### 4.2 Resectability criteria

The Beyond TME collaboration considers poor performance status, bilateral sciatic nerve encasement, and circumferential bone involvement as absolute contraindications to curative-intent surgery, with poor performance status referring to patients considered medically unfit to tolerate major pelvic surgery. Tumor extension through the sciatic notch, high sacral involvement, and the need of en bloc resection or reconstruction of the external iliac vessels are deemed relative contraindications. Nevertheless, it is noted that selected patients managed by highly specialized centers and multidisciplinary teams may still benefit from bTME surgery (4).

The eligibility criteria for curative-intent surgery in the included cohorts of this review are aligned with these principles. The main preconditions across all institutions were the probability of achieving negative margins (R0) and fair to good performance status. However, the definition of “absolute” or “relative” contraindications varied between centers (3, 10, 11).

This variability mirrors the lack of guidelines on the surgical management of RRRC and differences in surgical units or available resources.

### 4.3 Neoadjuvant therapy and re-irradiation strategies

The bTME consensus strongly recommends neoadjuvant CRT for patients with locally advanced rectal cancer and RRC, considering radiotherapy-naïve status and cumulative previously delivered dose with individualized radiotherapy planning. Additionally, the consensus underscores the potential benefit of IORT, stereotactic RT, and brachytherapy (4).

The reported neoadjuvant and intraoperative techniques of the current cohort review reflect the substantial heterogeneity among the included institutions. Each treatment plan was selectively driven by prior RT exposure, tumor distribution, and institutional experience or resource availability.

Given the paucity of high-level evidence, a cohort study analyzing re-recurrences of curative-intent treatment for RRC observed that almost half of failures occurred in the field of the previously selected target volume, thereby underscoring the need for optimized target volume delineation in re-irradiation management (13). Following that analysis, a subsequent prospective quality assurance study assessing the quality of delivered CRT within the patients of PelvEx II trial revealed that real time peer-review led to expansion of delineated gross and clinical target volumes (14).

Due to the heterogeneous application of IORT in RRRC across the included studies, the development of consensus-based criteria defining patient selection may be required. As underlined by Haddock et al., IORT has primarily been carried out as a selective dose-escalation strategy in margin-threatened or dose-limited settings in locally advanced rectal cancer and RRC, rather than as a standard component of neoadjuvant treatment (15).

### 4.4 R-margins and definition

Clear resection margins represent the main prognostic factor for patients undergoing bTME surgery, however no specific definitions for the desirable extent of R0 have been systematically proposed (4), as also shown by the presentation of R-margins in the included cohorts of this review (3, 10, 11). A retrospective, single center study tried to estimate the appropriate radial margins by comparing patients’ outcome with the achievable R-status. Specifically, the investigators grouped the patients into three groups; “wide; >1mm “, “ narrow; 0-1mm “, “ exposed; R1”. The conclusions were in line with the bTME consensus, indicating that circumferential margins <1 mm are a negative outcome predictor since the possibility of residual cancer cells (16).

Surgical planes in proximity to the area of recurrence in patients undergoing pelvic exenteration are often fibrotic due to prior RT; given the difficulty in predicting the exact extent of residual disease, wider circumferential resection margins are thereby warranted.

### 4.5 Common language – reporting standardization

The preoperative assessment of recurrence location using a predefined classification tool is paramount for intraoperative strategy planning. Pelvic MRI is the gold standard for the anatomical characterization of the tumor in relation to the pelvic fascial boundaries and prognostic factors such as extramural vascular invasion (EMVI) and nodal status. Over time, many study groups have proposed classification systems to stratify the extent of local recurrent disease based on the degree of symptoms, fixation, or invasion pattern (17). Recently, the bTME collaboration introduced a structured MRI-based classification tool for locally RRC, defining seven compartments formed by the intrapelvic fasciae that correspond to potential surgical resection planes (18).

The common key terminology should be encompassed into operative reports, specifying the exact pelvic organs removed. Due to high reporting heterogeneity, the Lexicon Collaboration mapped out a structured form denoting the extent of sacrectomy, lymphadenectomy, neurovascular ligation and excision of urogenital system or pelvic floor muscles (19). Although plastic reconstruction is recognized as a mandatory domain for reporting complex pelvic exenteration, Giannas E. et al. highlighted the necessity of precisely describing the type of reconstruction for re-recurrent rectal cancer. In previously bTME operated patients, conventional pedicled flaps may not be feasible, considering free-flaps or novel technique such as bowel friendly implants as alternative approaches (8).

RRRC cases are complex and require accurate MDT coordination, making the use of common terminology mandatory. This is reflected in the sparse reporting within our data regarding the location of re-recurrence, the extent of sacrectomy, and pelvic organ removal. In addition, only a few of the included cases mentioned the type of reconstruction, without clarifying the rationale for the selected flap or the presence of a previous flap.

Standardized radiological and surgical reporting may improve decision-making and outcome comparability in RRRC.

### 4.6 Limitations

The available evidence was limited to retrospective observational studies with relatively small sample sizes and potential referral bias, selection bias, and institutional expertise bias, as all included cohorts originated from highly specialized referral centers. Furthermore, substantial heterogeneity existed in patient selection, therapeutic strategies, and institutional protocols, spanning nearly three decades during which imaging modalities and perioperative management evolved significantly. In addition, not all centers had access to the same therapeutic resources (e.g., intraoperative radiotherapy or advanced reconstructive techniques), further contributing to variability in management. Moreover, oncological outcomes, including overall survival, were reported at inconsistent time points across the included studies, without standardized statistical measures such as confidence intervals or variance estimates. Quality-of-life outcomes were inconsistently reported or absent across the included studies, precluding assessment of postoperative morbidity, urinary and sexual dysfunction, wound-related complications, and patient-reported outcomes. These factors precluded quantitative synthesis or meta-analysis and limited the overall certainty of conclusions, restricting the generalizability of the findings.

## 5 Conclusion

Curative-intent surgery for RRRC is feasible in highly selected patients in specialized multidisciplinary centers. Available evidence consistently suggests that achieving clear resection margins is the most important prognostic factor associated with oncological outcomes. However, due to the high heterogeneity in adjunct perioperative treatment strategies, including MRI-based compartment classification, operative reporting (such as Lexicon reporting framework), and margin definitions, as well as the limited number of small retrospective series, the development of standardized frameworks and consensus-based recommendations is required to optimize multidisciplinary treatment planning. Future prospective multicenter studies or international registries are required to establish evidence-based management strategies for RRRC.

## Data Availability

The original contributions presented in the study are included in the article/**Supplementary Material**. Further inquiries can be directed to the corresponding author.
