# Supplementary material for: Current evidence on the management of re-recurrent rectal cancer: a systematic review
Source: Front Oncol. 2026 Jul 15;16:1867185. doi: 10.3389/fonc.2026.1867185 (PMC13414183; doi:10.3389/fonc.2026.1867185)
Supplement: Supplementary file 1 [file Table1.docx]

**Supplementary Tables**

**Table S1.** Search terms employed to screen different electronic databases for the literature search.

| **Database** | **Search terms** |
| --- | --- |
| PubMed | ("rectal cancer"[Title/Abstract] OR "rectal carcinoma"[Title/Abstract] OR "rectal neoplasm"[Title/Abstract] OR "rectal neoplasms"[Title/Abstract] OR "rectal adenocarcinoma"[Title/Abstract]) AND ("re-recurrence"[Title/Abstract] OR "re-recurrent"[Title/Abstract] OR "second recurrence"[Title/Abstract] OR "2nd recurrence"[Title/Abstract] OR "re-relapse"[Title/Abstract] OR "local re-recurrence"[Title/Abstract] OR "locally re-recurrent"[Title/Abstract]) AND ("re-surgery"[Title/Abstract] OR "re-resection"[Title/Abstract] OR "salvage surgery"[Title/Abstract] OR "re-irradiation"[Title/Abstract] OR "repeat radiotherapy"[Title/Abstract] OR "re-chemotherapy"[Title/Abstract] OR "repeat chemotherapy"[Title/Abstract] OR "re-challenge chemotherapy"[Title/Abstract] OR "immunotherapy"[Title/Abstract] OR "immune checkpoint inhibitor"[Title/Abstract] OR "multimodal therapy"[Title/Abstract] OR "treatment"[Title/Abstract] OR "management"[Title/Abstract] OR "therapy"[Title/Abstract] OR "therapeutic strategy"[Title/Abstract]) |
| Scopus | TITLE-ABS-KEY(“rectal cancer” OR “rectal carcinoma” OR “rectal neoplasm” OR “rectal neoplasms” OR “rectal adenocarcinoma”) AND TITLE-ABS-KEY(“re-recurrence” OR “re-recurrent” OR “second recurrence” OR “2nd recurrence” OR “re-relapse” OR “local re-recurrence” OR “locally re-recurrent”) AND TITLE-ABS-KEY(“re-surgery” OR “re-resection” OR “salvage surgery” OR “re-irradiation” OR “repeat radiotherapy” OR “re-chemotherapy” OR “repeat chemotherapy” OR “re-challenge chemotherapy” OR “immunotherapy” OR “immune checkpoint inhibitor” OR “multimodal therapy” OR “treatment” OR “management” OR “therapy” OR “therapeutic strategy”) |
| Cochrane Library | ("rectal cancer" OR "rectal carcinoma" OR "rectal neoplasm" OR "rectal neoplasms" OR "rectal adenocarcinoma") AND ("re-recurrence" OR "re-recurrent" OR "second recurrence" OR "2nd recurrence" OR "re-relapse" OR "local re-recurrence" OR "locally re-recurrent") AND ("re-surgery" OR "re-resection" OR "salvage surgery" OR "re-irradiation" OR "repeat radiotherapy" OR "re-chemotherapy" OR "repeat chemotherapy" OR "re-challenge chemotherapy" OR "immunotherapy" OR "immune checkpoint inhibitor" OR "multimodal therapy" OR "treatment" OR "management" OR "therapy" OR "therapeutic strategy") |
| Web of Science | TS=(“rectal cancer” OR “rectal carcinoma” OR “rectal neoplasm” OR “rectal neoplasms” OR “rectal adenocarcinoma”) AND TS=(“re-recurrence” OR “re-recurrent” OR “second recurrence” OR “2nd recurrence” OR “re-relapse” OR “local re-recurrence” OR “locally re-recurrent”) AND TS=(“re-surgery” OR “re-resection” OR “salvage surgery” OR “re-irradiation” OR “repeat radiotherapy” OR “re-chemotherapy” OR “repeat chemotherapy” OR “re-challenge chemotherapy” OR “immunotherapy” OR “immune checkpoint inhibitor” OR “multimodal therapy” OR “treatment” OR “management” OR “therapy” OR “therapeutic strategy”) |
